# Selective multi-nanosoldering for fabrication of advanced solution-processed micro/nanoscale metal grid structures

**DOI:** 10.1038/s41598-020-63695-0

**Published:** 2020-04-22

**Authors:** Y. S. Oh, J. Lee, D. Y. Choi, H. Lee, K. Kang, S. Yoo, I. Park, H. J. Sung

**Affiliations:** 1 Department of Mechanical Engineering, KAIST, 291 Daehak-ro, Yuseong-gu Daejeon, 34141 Korea; 2 School of Electrical Engineering, KAIST, 291 Daehak-ro, Yuseong-gu Daejeon, 34141 Korea

**Keywords:** Mechanical engineering, Solar cells

## Abstract

Solution-processed metal grid transparent conductors with low sheet resistance, high optical transmittance and good mechanical flexibility have great potential for use in flexible optoelectronic devices. However, there are still remaining challenges to improve optoelectrical properties and electromechanical stability of the metallic structures due to random loose packings of nanoparticles and the existence of many pores. Here we introduce a selective multi-nanosoldering method to generate robust metallic layers on the thin metal grid structures (< a thickness of 200 *n*m), which are generated *via* self-pining assisted direct inking of silver ions. The selective multi-nanosoldering leads to lowering the sheet resistance of the metal grid transparent conductors, while keeping the optical transmittance constant. Also, it reinforces the electromechanical stability of flexible metal grid transparent conductors against a small bending radius or a repeated loading. Finally, organic light-emitting diodes based on the flexible metal grid transparent conductors are demonstrated. Our approach can open a new route to enhance the functionality of metallic structures fabricated using a variety of solution-processed metal patterning methods for next-generation optoelectronic and micro/nanoelectronic applications.

## Introduction

Transparent conductors (TCs) are essential components for a variety of optoelectronic devices, including organic solar cells^1^, organic light-emitting diodes (OLEDs)^2^, touch screen panels^3^. Recently, there have been increasing efforts in developing alternative nanomaterial TCs based on carbon nanotubes^4^, graphene^5^, metal nanowires^6^ and metal grids^7^ to replace indium tin oxide (ITO)-based films. Among these nanomaterial-based TCs, the metal grid TCs have been spotlighted for use in flexible optoelectronic devices due to facile control over their grid width and spacing, scalability to large-area application, uniform sheet resistance and low junction resistance^8–11^. Especially, the metal grid TCs are typically fabricated using a variety of printing technologies of metal nanoparticles (NPs), including inkjet printing^11^, gravure printing^12^, micro-contact printing^13^ and direct imprinting^14–16^ at low costs and in a high throughput manner.

The solution-processed metal grid TCs show the electrical conductivity from merging of neighboring NPs and eliminating of insulating organic complexes by several sintering processes, including thermal sintering^17^, photonic sintering^18^, laser sintering^19^ and chemical sintering^20^. They exhibited the poor electrical conductivity compared with the evaporative metal grids obtained using photo-roll lithography^21–23^ due to random loose packings of NPs, incomplete elimination of organic complexes and the existence of many pores between NPs^24,25^. Oh *et al*.^16^ carried out experiments on temperature-controlled direct imprinting of silver ionic ink, enhancing the optoelectrical properties of metal grid TCs by the porosity reduction. The unsintered metal grid structures with non-uniform shape and thickness (> μm) suffered from a local damage or a crack generation, resulting in locally unstable mechanical, electrical and optical properties during the mold detachment. Especially, these issues hindered the use of large-scale TCs.

Recently, solution-grown (SG) metal grid TCs fabricated using electroless plating or electroplating have been reported to show excellent optoelectrical properties. Sciacca *et al*.^26^ introduced the SG metal grid TCs generated using electroless plating and soft lithography on a glass substrate. After rapid thermal annealing, the SG metal grid TCs showed superior optoelectrical properties than those obtained using vacuum-based metal deposition: sheet resistance (*R*_s_) of 3.5 Ω sq^−1^ vs 10.7 Ω sq^−1^ at transmittance (*T*_550nm_) of 76%, respectively. Jin *et al*.^27^ suggested a method for directly generating the SG metal grid TCs on polyethylene terephthalate (PET) substrates, which were coated using a poly(dopamine) to improve interfacial adhesion between the SG metal NPs and the substrate. The SG metal grid TCs exhibited *R*_s_ of 8 Ω sq^−1^ at *T*_550nm_ of 91% and a reasonable electromechanical stability under bending stresses. Also, Khan *et al*.^28^ showed the metal grid structures that are embedded and mechanically anchored onto a flexible substrate using electroplating process. This method could provide flexible metal grid TCs with the high aspect ratio, showing *R*_s_ < 1 Ω sq^−1^ at *T*_550nm_ = 91%. However, all of these methods suffered from a time-consuming and expensive task related to the generation of polymer masks and the need of dry (or wet) etching process.

Here we introduce a selective multi-nanosoldering (SMN) method for simultaneously enhancing both optoelectrical properties and electromechanical stability of solution-processed metal grid structures without polymer masks and etching process. The thin metal grid structures (< a thickness of 200 *n*m) can be fabricated using a self-pinning assisted direct inking of silver ions based on the ink capturing. When the mold is detached in a liquid state of ink, the process facilitates the prevention of unwanted structural damages from the metal grids. After the SMN treatment, the metal grid structures based on loosely packed metal NPs were transformed into compact and robust metallic structures. The effect of SMN on the optoelectrical properties of the metal grid TCs was evaluated by considering cycle number, growth time (*t*_G_), growth temperature (*T*_G_), sintering temperature (*T*_S_). Also, the effect of SMN on the electromechanical stability of flexible metal grid TCs after a transfer process was examined under static and dynamic bending stresses.

## Results

### Self-pinning assisted direct inking of silver ions

**Figure 1:**
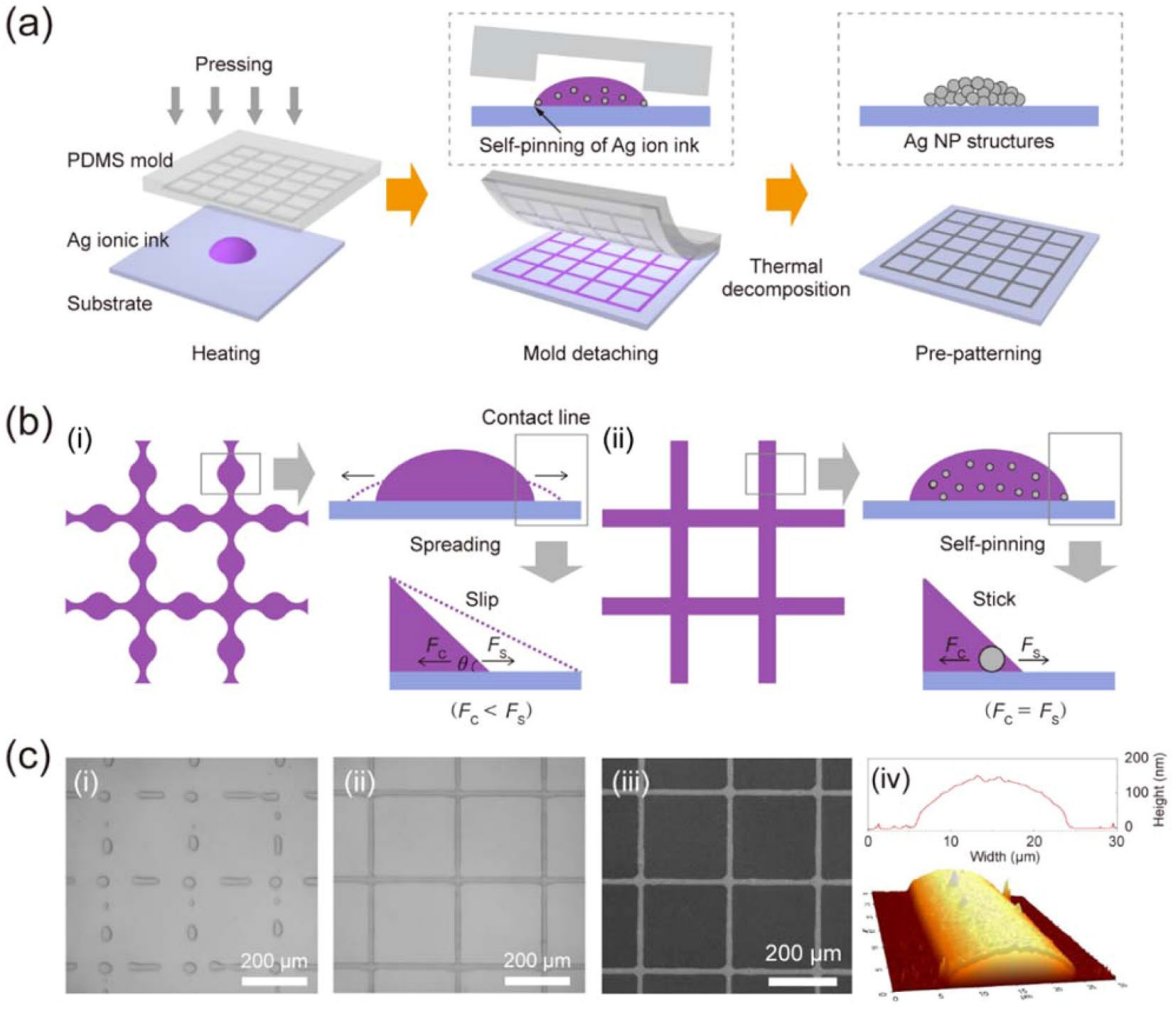
Schematic illustration of self-pinning assisted direct inking of Ag ions for fabrication of micro/nanoscale metallic structures. (**a**) Fabrication of metal grid TCs using the self-pinning assisted direct inking of silver ions. (**b**) Mechanism of (i) withdrawing behavior and (ii) self-pinning behavior of silver ionic ink after mold detachment. (**c**) Microscope images of (i) spreading/dewetting and (ii) self-pinning of silver ionic ink; (iii) SEM image of metal grid TCs after the thermal decomposition of silver ions. (iv) AFM surface profile and image of metal grid line.

The metal grid structures were fabricated on a glass substrate *via* self-pinning assisted direct inking of silver ions. A schematic illustration of the process is shown in Fig. 1a. Note that the process directly generates a grid-patterned ink, rather than metal NP structures. A silver ionic ink was captured within a grid-patterned mold under heating at 60 °C and pressing at 130 kPa. During solvent evaporation, some of the silver ions were thermally decomposed to silver NPs and organic complexes. After the mold was detached, the meniscus of the grid-patterned ink was kept pinned at contact lines due to the spreading inhibition induced by the confinement of the silver NPs and organic complexes^29^. The grid-patterned ink was thermally decomposed at an elevated temperatures (250 °C) to form the silver NP structures and to eliminate of larger amounts of organic insulating complexes, which cause the generation of many pores inside the metal NP structures^16^.

Figure 1b shows a schematic illustration of spreading and self-pinning mechanism of the grid-patterned ink. The spreading behavior of a liquid droplet on substrates is natural phenomenon. In general, it is described in terms of the spreading coefficient (*S*) that is given by *S* = *σ*_sg_ − *σ*_sl_ − *σ*_lg_, where *σ* is the interfacial tension between two different phases, including solid-gas (*σ*_sg_), solid-liquid (*σ*_sl_) and liquid-gas (*σ*_lg_), respectively. When *S* < 0, an equilibrium force balance at contact lines is described by Young’s equation, given by *σ*_sg_ = *σ*_sl_ + *σ*_lg_cos *θ*_E_, where *θ*_E_ is an equilibrium contact angle. However, *θ*_E_ of the liquid droplet was modulated by the NPs confined at contact lines. Weon *et al*.^29^ suggested a simple model to quantitatively describe the self-pinning behavior of colloidal droplet by equating a spreading force (*F*_s_ = 2π*RS*) with a capillary force (*F*_c_ = 2π*rN*σ_lg_ (cos*θ*)^2^) at contact lines, where *R* is the diameter of droplet, *r* is the average diameter of NPs, *N* is the number of NPs at contact lines and *θ* is the contact angle, respectively. They insisted that the self-pinning behavior of the colloidal droplet should be generated at a critical value of 15° and a critical linear packing fraction of 10%, regardless of the NP size and the initial volume fraction.

Similar to the colloidal droplet, *θ*_E_ of a silver ionic ink droplet was modulated from the thermally decomposed silver NPs and organic complexes confined at contact lines. Figure S1a shows photographic images for the measured *θ*_E_ of the ink droplet after ink addition and heating. An ink volume of 5 μl was added to the ink droplet (5 μl). The measured *θ*_E_ of the ink droplet without heating showed similar (or slightly decreased) values due to solvent evaporation. After heating at 60 °C for 2 min, the ink droplet showed the spreading behavior at *θ*_E_ = 50°. The measured *θ*_E_ of the ink droplet increased with an addition of ink due to the thermal reduction of silver ions. The measured *θ*_E_ of the ink droplet slowly decreased with solvent evaporation. This self-pining behavior is important to guarantee the physical continuity and the rigorous resolution of the grid-patterned ink as well as the capture of large amounts of ink.

The self-pinning of the grid-patterned ink was easily controlled by a heating time at a fixed temperature of 60 °C to minimize unwanted residual layers within grid spacings. In Fig. 1c(i-ii), microscope images show the spreading and self-pinning behavior of the grid-patterned ink after two heating times of 30 and 300 sec, respectively. Figure 1c(i) shows that the continuous grid-patterned ink was divided into irregular droplets, so called dewetting behavior, due to the spreading behavior of the ink and its surface instability. The silver NP structures, generated from the broken droplets, did not have the electrical conductivity regardless of the sintering process. Figure 1c(ii) shows that the grid-patterned ink was kept to an original shape due to the spreading inhibition of confined NPs at contact lines. In Fig. S2a, a wetting behavior of silver ionic ink after the mold detachment was compared at a heating time. After the thermal decomposition and sintering process, the metal grid TCs showed reasonable optoelectrical properties, which is approximately *R*_s_ of 20 Ω sq^−1^ at *T*_550nm_ of 88.0%. In Fig. S2b, *R*_s_ and *T*_550nm_ of the metal grid TCs for different heating times were compared. Figure 1c(iv) shows an atomic force microscopy (AFM) surface profile and an AFM image of the metal line structures with an average width of 17 μm and a maximum height of 151.4 nm.

### Effect of SMN on optoelectrical properties

**Figure 2:**
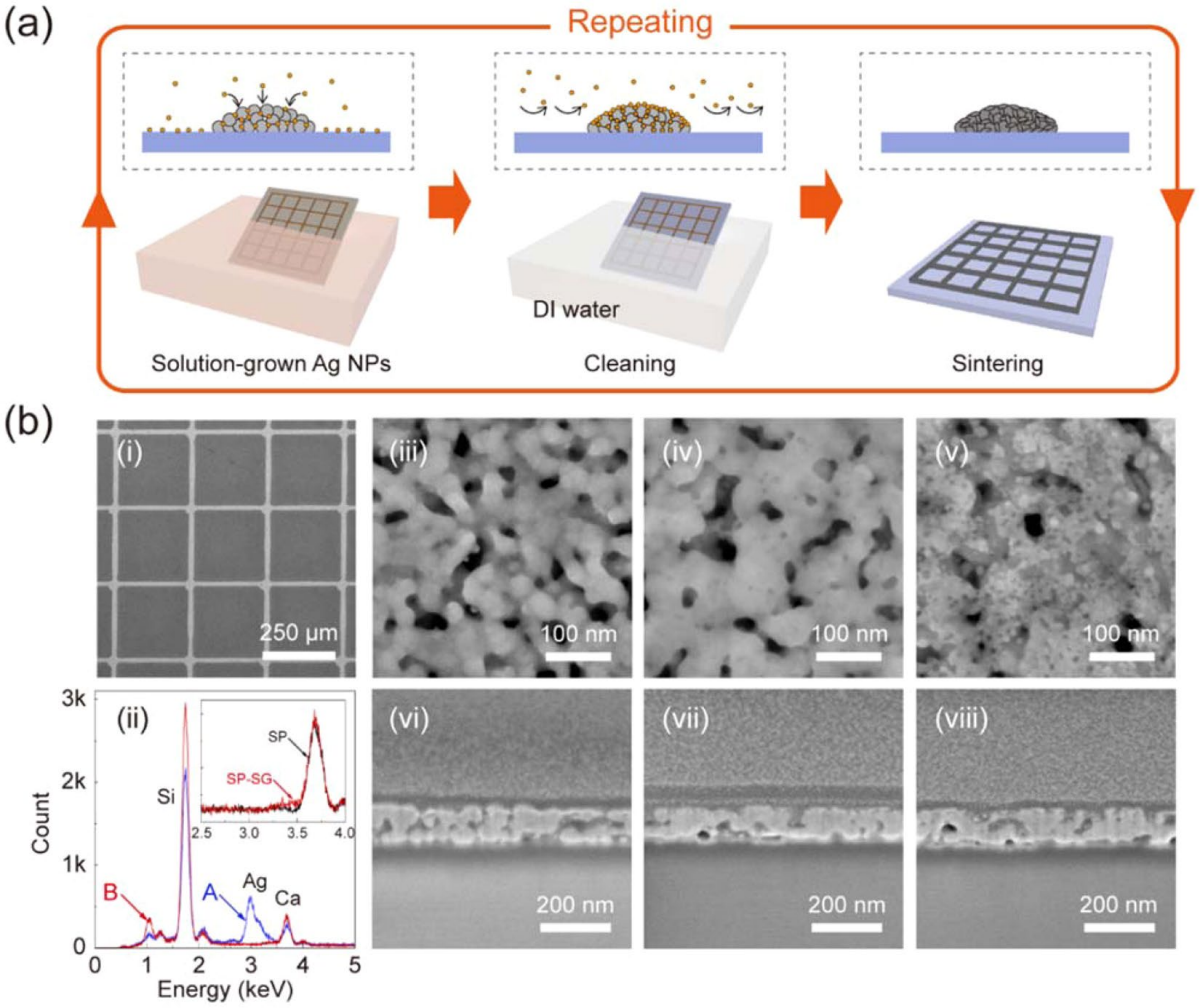
Effect of SMN on optoelectrical properties of the metal grid structures. (**a**) Schematic illustration of SMN. (**b**) (i) SEM images of the SG metal grid structures; (ii) EDX spectra taken from the grid line (A) and spacing (B) of the SG metal grid structures. The inset shows magnified spectra of EDX taken from (B), respectively; (iii-iv) SEM images of top surface of the metal grid structures before and after SMN 1 of 2 and 4 cycles, respectively; (**c**) Cross-sectional FIB-SEM images of the meal grid structures before and after SMN 1 of 2 cycles and 4 cycles, respectively.

As the metal grid structures include random loose packings of silver NPs with many pores, they usually have larger electrical resistivity (~1.50 × 10^−7^ Ω m) than that of bulk silver (1.59 × 10^−8^ Ω m). Especially, when the size of the metal grid structures decreases to micro/nanoscale, the resistivity can be considerably increased and locally unstable due to electron scattering at grain boundary of loosely packed NPs^26,30^. Figure 2a shows a schematic illustration of SMN process transforming random loose NP packings of surface of metal grids into robust metallic structures. The SMN method is based on a repetitive process of selective nanosoldering (SN), which consists of solution-growth of silver NPs on the metal grids by electroless plating, elimination of residual silver NPs within grid spacings by cleaning process, and merging of the SG silver NPs and the metal grid structures by thermal sintering. Note that the metal grid TCs treated using SMN or SN are defined as the SG metal grid TCs. Figure 2b(i) shows a scanning electron microscope (SEM) image of the SG metal grid structures after SMN of 4 cycles for *t*_G_ = 1 min (SMN 1). The SG silver NPs were deposited on the metal grid TCs with minimum residual layers within grid spacings, and then were merged at 250 °C for 1 min. In Fig. 2b(ii), energy dispersive X-ray analysis was taken from the grid line (A) and grid spacing (B) of the SG metal grid TCs after SMN 1, respectively. The inset shows magnified spectra of EDX taken from the grid spacing (B) of the metal grid TCs and SG metal grid TCs, respectively. A silver peak in the spectrum of B obtained using EDX was not observed. This result means that the SG silver NP-based residual layers within grid spacings is negligible. Figure 2b(iii–v) shows the SEM images of the top surface of the metal grid structures, and the SG metal grid structures after SMN of 2 and 4 cycles for 1 min, respectively. The SG silver NPs were merged with loosely packed metal NPs presented on the top surface of the metal grid structures. After SMN 1, the top surface of the SG metal grid structures was changed to more compact and denser metallic structures. Figure 2b(vi–viii) shows the cross-sectional focused ion beam (FIB)-SEM images of the metal grids, and SG metal grids after SMN of 2 and 4 cycles for 1 min, respectively. In Fig. 2b(vi), the metal grid structures exhibited many grain boundaries of NPs and pores, which are main sources of the poor electrical resistivity of metal structures. After SMN, the SG metal grid structures showed more interconnected and continuous connection of metal NPs in Fig. 2b(vii) and (viii).

**Figure 3:**
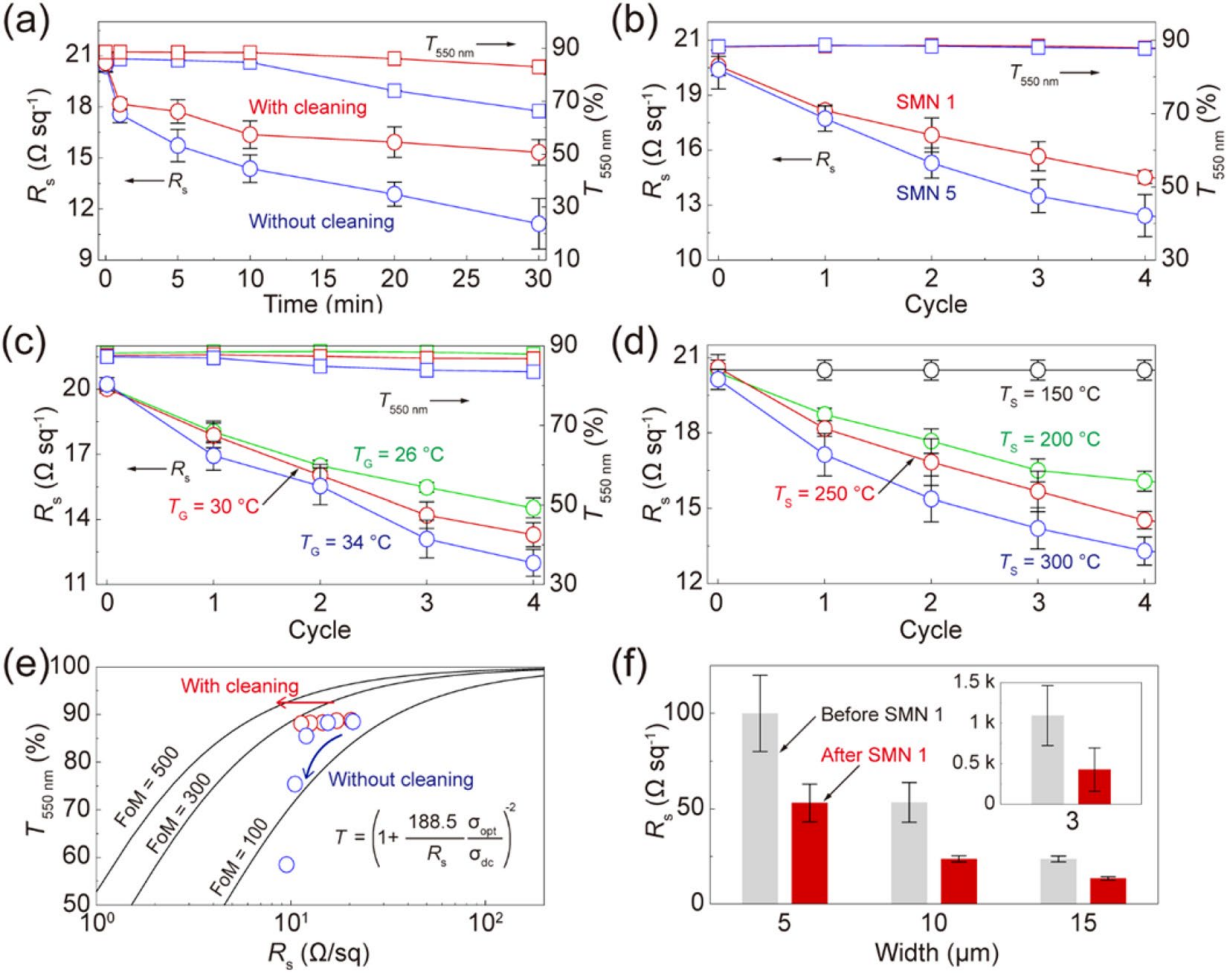
Effect of SMN on optoelectrical properties of micro/nanoscale metallic structures. (**a**) Sheet resistances and transmittances of the SG metal grid TCs treated using SMN and multi-nanosoldering, respectively. (**b**) Sheet resistances and transmittances of the SG metal grid TCs. (**c**) Sheet resistances and transmittances of the SG metal grid TCs at different growth temperatures. (**d**) Sheet resistances and transmittances of the SG metal grid TCs treated using SMN 1 at different sintering temperatures. (**e**) FoM values of the SG metal grid TCs according to SMN vs multi-nanosoldering. (**f**) Sheet resistances of the metal grid TCs and SG metal grid TCs with different widths according to SMN 1, respectively.

In SMN, the cleaning process played a critical role in decreasing the SG silver NP-based residual layers within grid spacings, while it led to gradually decreasing *R*_s_ of the SG metal grid TCs due to a slight loss of the SG silver NPs on the metal grid structures. In Fig. 3a, *R*_s_ and *T*_550nm_ of the SG metal grid TCs treated using SN for *t*_G_ = 1 to 30 min were compared by considering the cleaning process. For SN within *t*_G_ of 10 min, *R*_s_ of the SG metal grid TCs decreased with keeping *T*_550nm_ a constant. Figure 3b shows *R*_s_ and *T*_550nm_ of the SG metal grid TCs during SMN 1. *R*_s_ of the metal grid TCs decreased from 20.6 to14.5 Ω sq^−1^ over *T*_550nm_ of 87.9%. When *t*_G_ increased to 5 min, *R*_s_ of the metal grid TCs decreased to 12.4 Ω sq^−1^ over *T*_550nm_ of 87.8%. In Figure S3a and b, transmittance spectra of the SG metal grid TCs treated using SMN 1 and 5 are shown. During SMN, a significant reduction in the spectral intensity over a wide wavelength range was not observed. A slight reduction in the spectral intensity near wavelength of 400 nm was generated due to residual metal NPs^9^. Transmittance spectra of the SG metal grid TCs treated using a multi-nanosoldering are compared, as shown in Figure S3c and d. After multi-nanosoldering of several cycles, a significant reduction in the spectral intensity over a wide wavelength range was observed. It is caused by an incomplete elimination of the remaining NP and solution within grid spacings.

Although an increase of *t*_G_ led to sharply decreasing *R*_s_ of the SG metal grid TCs, it required a considerable processing time. Instead of SMN for long *t*_G_, *T*_G_ was used to increase the solution-growth rate of metal NPs. Figure 3c shows *R*_s_ and *T*_550nm_ of the SG metal grid TCs treated using SMN 1 at different values of *T*_G_. When *T*_G_ increased from 26 °C to 30 °C, the SMN for 1 min facilitated a considerable decrease in *R*_s_ of the SG metal grid TCs with less than 1% of *T*_550nm_. The SMN 1 treated at *T*_G_ = 34 °C led to sharply decreasing *R*_s_ of the SG metal grid TCs, while it sacrificed approximately 4% of *T*_550nm_. This result means that large amounts of the SG metal NPs, rapidly generated at the increased *T*_G_, could be insufficiently eliminated during the cleaning process. Also, *R*_s_ of the SG metal grid structures was compared with controlling *T*_S_ after the solution-growth of metal NPs. Figure 3d shows *R*_s_ of the SG metal grid TCs generated using SMN 1 at different values of *T*_S_ ranged from 150 to 300 °C. *R*_s_ of the SG metal grid TCs did not decrease at *T*_S_ = 150 °C. However, when *T*_S_ increased from 200 °C to 300 °C, *R*_s_ of the SG metal grids rapidly decreased to lower values.

Optoelectrical properties of the SG metal grid TCs could be evaluated using the following equation; *T* = (1 + 188.5(σ_Op_/σ_DC_)/*R*_s_)^−2^, where *σ*_Op_ and *σ*_DC_ are the optical and direct current conductivity of the nanostructure-based TCs, respectively. The term *σ*_DC_/*σ*_Op_ is used as a figure of merit (FoM), whose larger value represents better TC performance. Figure 3e shows FoM values of the SG metal grid TCs according to SMN 5. The FoM value of the SG metal grid TCs increased from 150 to 252 by SMN. On the other hands, multi-nanosoldering for 5 min without the cleaning increased the FoM value to 193, and then decreased to 19 due to a significant loss of *T*_550nm_. Figure 3f shows *R*_s_ and *T*_550nm_ of the SG metal grid TCs with different widths after SMN 1. When a linewidth decreased with keeping *T*_550nm_ of 90% by a geometrical consideration, the metal grid TCs showed the higher sheet resistance due to electronic scattering and local defects. Especially, the metal grid TCs with a linewidth of 3 μm showed *R*_S_ of ~kΩ, which lowered to few hundred Ω after SMN 1. This result means that the SMN treatment leads to enhancing optoelectrical properties of micro/nanoscale metallic structures fabricated using a variety of solution-based patterning processes.

### Effect of SMN on electromechanical stability

**Figure 4:**
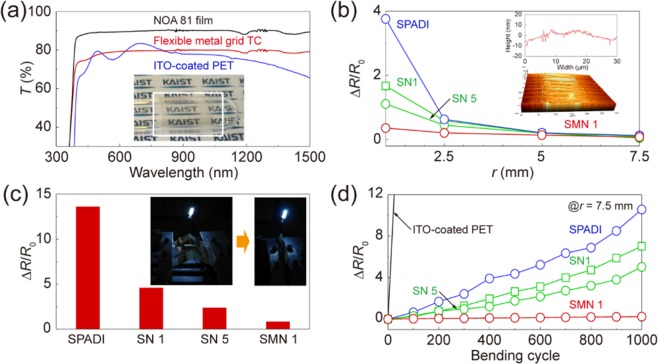
Effect of SMN on electromechanical stability of the metal grid TCs. (**a**) Transmittance spectra over a wavelength range of 350–1500 nm, of the NOA 81 film, the flexible SG metal grid TCs (SMN 1), and the ITO-coated PET film, respectively. The inset shows a photograph of the flexible SG metal grid TCs on a test tube. (**b**) Normalized resistance change of the flexible metal grid TCs as a function of bending radius, respectively. The inset shows an AFM image of the embedded grid line for flexible metal grid TCs. (**c**) Normalized resistance change of the flexible metal grid TCs under folding, respectively. The inset shows photographs of the flexible metal grids under folding. (**d**) Normalized resistance change of the flexible metal grid TCs under repeated bending, respectively.

The electromechanical stability of metal grid structures against bending stresses is one of the most important issues for use in next-generation flexible optoelectronic devices. The SG metal grid TCs were transferred, and embedded into transparent and UV-curable polymer films of Norland Optical Adhesive 81 (NOA 81) to evaluate the electromechanical stability. In general, a transfer process of the metallic TCs leads to lowering a surface roughness, to giving a flexibility and to protecting them from mechanical stress and surface oxidation. After the transfer process, a transmittance spectrum of the flexible SG metal grid TCs was compared with those obtained from a commercially available ITO-coated PET film in Fig. 4a. A transmittance spectrum of bare NOA 81 film was shown as a reference. It should be noted that the transmittance spectra through the substrate are measured. Optoelectrical properties of the flexible SG metal grid TCs (*R*_s_ of 13.1 Ω sq^−1^ and *T*_550 nm_ of 78.8%) showed superior than those obtained using the ITO-coated PET film (*R*_s_ of 15.1 Ω sq^−1^ and *T*_550 nm_ of 75.4%). The transmittance spectrum of the ITO-coated PET film decreased with significant fluctuations over the wavelength range of 400–1500 nm. On the other hand, the transmittance spectrum of the flexible SG metal grid TCs remained constant. The inset shows a photograph of the SG metal grids TCs attached on a test tube, demonstrating its flexibility and transparency.

Figure 4b plots ∆*R*/*R*_o_ obtained from the flexible metal grid TCs (no treatment, SN 1, SN 5, SMN 1 of 4 cycles, respectively) under static bending stresses. The inset shows an AFM surface profile and an AFM image of the flexible SG metal grid TCs where the metal grid structures were embedded into the NOA 81 film with a root-mean-square surface roughness of 4.3 nm and a maximum peak-to-valley value of 22.9 nm. All of the flexible metal grid TCs (*R*_o_ = 23.5, 20.2, 16.6 and 13.4 Ω, respectively) showed superior electromechanical stability than the ITO-coated PET film under static bending stresses. However, *R*/*R*_o_ of the flexible metal grid TCs significantly increased at *r* ≤ 2.5 mm due to serious damage. *R*/*R*_o_ of the flexible metal grid TCs treated using SN decreased with increasing *t*_G_ at *r* = 1 mm. The flexible metal grid TCs treated using SMN 1 showed lower *R*/*R*_o_ than those obtained using both SN 1 and SN 5. When the flexible metal grid TCs were bent up to *r* = 1 mm, the values of Δ*R*/*R*_o_ increased by 376% and 36%, respectively. Also, ∆*R*/*R*_o_ of the flexible metal grid TCs were evaluated under an extremely localized bending stress that is so called folding. Figure 4c shows ∆*R*/*R*_o_ of the flexible metal grid TCs under folding (*r* < 0.3 mm), respectively. The metal grid TCs underwent a ∆*R*/*R*_o_ of more than 1360%. The flexible metal grid TCs treated using SN 1 and SN 5 showed a considerable decrease of *R*/*R*_o_ with increasing *t*_G_ (∆*R*/*R*_o_ of less than 460% and 240%, respectively). The flexible SG metal grid TCs treated using SMN 1 showed ∆*R*/*R*_o_ of less than 85%. The inset shows a photographic image of LED integrated circuit operation connected with the flexible SG metal grid TCs under folding. This result reveals that the SMN treatment leads to enhancing the electromechanical stability of the flexible metal grid TCs even under an extremely localized bending stress.

Figure 4d plots ∆*R*/*R*_o_ obtained from the flexible metal grid TCs (no treatment, SN 1, SN 5 and SMN 1 of 4 cycles, respectively) under dynamic bending stresses with *r* = 7.5 mm. Δ*R*/*R*_o_ of the ITO-coated PET film was evaluated as a reference. Although all of the flexible metal grid TCs showed similar values of ∆*R*/*R*_o_ under static bending stresses with *r* = 7.5 mm, they exhibited different aspects of ∆*R*/*R*_o_ under dynamic bending stresses. After 1000 cycles of repeated bending/relaxation, ∆*R*/*R*_o_ obtained from the flexible metal grid TCs increased by more than 1000%. The flexible metal grid TCs treated using SN 1 and SN 5 showed a considerable decrease of ∆*R*/*R*_o_ with increasing *t*_G_ (∆*R*/*R*_o_ of l than 700% and 500%, respectively). On the other hand, the flexible metal grid TCs treated using SMN 1 showed ∆*R*/*R*_o_ of less than 30%. The SMN treatment results in reinforcing the mechanical strength of the metallic structures, which leads to enhancing the electromechanical stability under static bending, folding and dynamic bending stresses.

### Applications to flexible OLEDs

**Figure 5:**
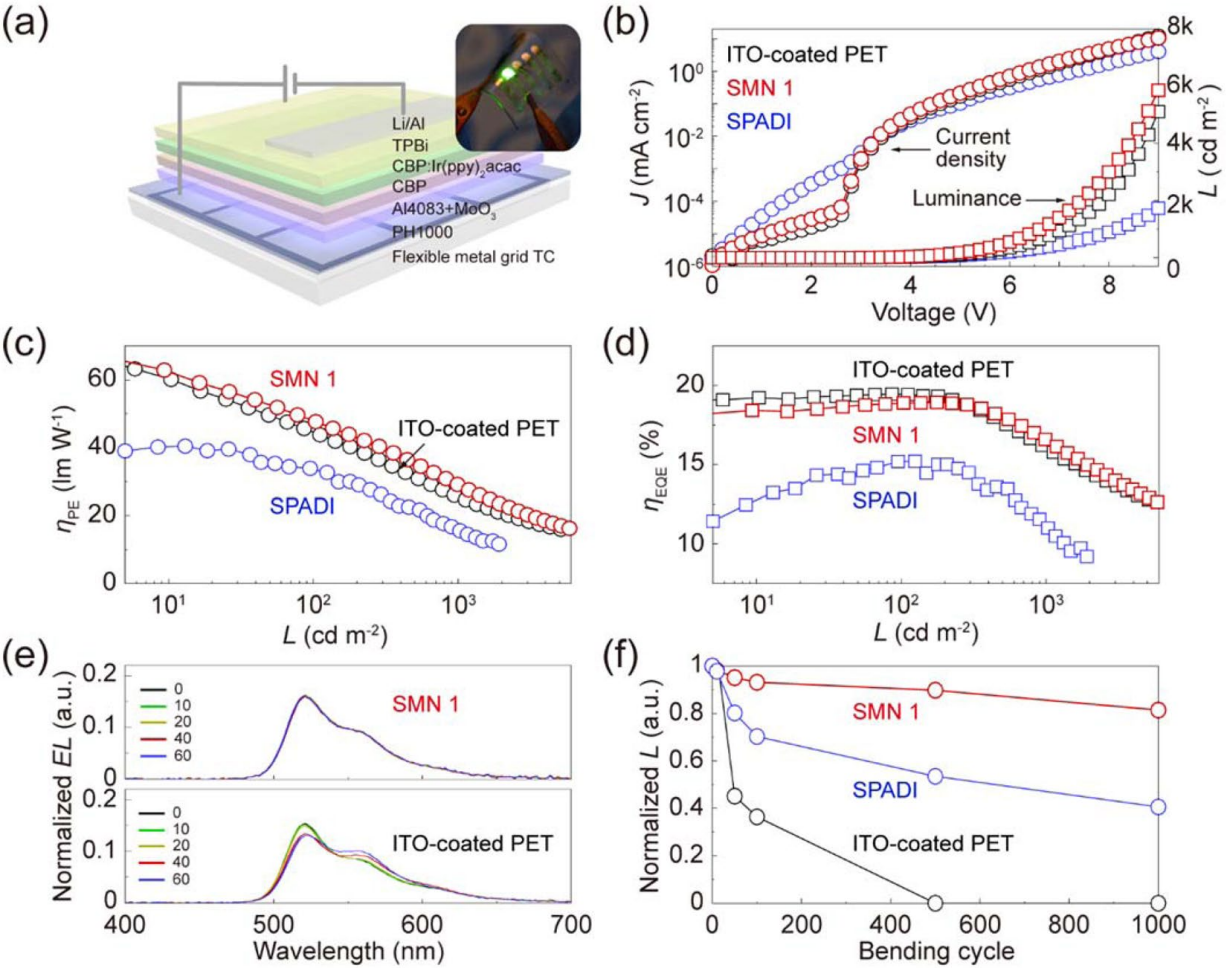
Fabrication of flexible OLEDs. (**a**) Schematic illustration of the OLEDs prepared using the flexible metal grid TCs. (**b**) Current density−voltage characteristics and luminance-voltage characteristics of the OLEDs based on the flexible metal grid TCs and ITO-coated PET, respectively. (**c**) Power efficiency of the flexible OLEDs. (**d**) External quantum efficiency of the flexible OLEDs. (**e**) Normalized EL intensity of the flexible OLEDs over a wavelength range of 400–700 nm. (**f**) Normalized luminance of flexible OLEDs as a function of bending cycles at *r* = 3.4 mm.

Using the flexible metal grid TCs, the green phosphorescent OLEDs were fabricated as shown in Fig. 5a. The highly conductive PEDOT:PSS (PH 1000 with 5 wt% of DMSO, 50 nm, 242 Ω sq^−1^) was used as a buffer transparent electrode on top of the flexible metal grid TCs. The low conductive PEDOT:PSS (PVP AI4083, 50 nm) and MoO_3_ were used as a hole injection layer^31^. On top of the MoO_3_, organic layers and metal electrode were sequentially deposited using thermal evaporator, and the detailed configuration is described in the experimental section. The flexible metal grid TCs-based OLEDs exhibited superior electro-luminous properties than those obtained using the flexible metal grid TCs due to excellent optoelectrical properties. Also, since the flexible metal grid structures is relatively compact and robust, the flexible metal grid TCs-based OLEDs showed leakage-free performances due to a minimal breakage during the transfer process. On the other hand, the flexible metal grid TCs-based OLEDs showed a large leakage current due to a breakage of the loose and weak metal grid structures during the transfer process that leads to rough bottom surface of the device (seen below 3 V in Fig. 5b). For this reason, the proposed device exhibited external quantum efficiency (*η*_EQE_) and power efficiency (*η*_PE_) as high as 18.9%, and 47.6 lm/W at 103.7 cd/m^2^, while the flexible metal grid TCs-based OLEDs showed the limited performance as low as 15.2% and 33.8 lm/W at 95.0 cd/m^2^, as shown in Figs. 5c and d. Figure 5e shows electroluminescence (EL) characteristics of the proposed and ITO-coated device. The proposed devices show the color stable performance with respect to viewing angle, while the devices based on ITO-coated PET exhibit the weak angular color shift due to its high refractive index that is prone to make weak microcavity effects in the device^32^. Moreover, the flexible OLEDs was evaluated to verify performance stability under dynamic bending tests with *r* = 4.3 mm. As a result, the flexible metal grid TCs-based OLEDs (tensile strain (ε_*f*_) of 1.97%, d_Sub-NOA_: 170 μm) shows relatively superior luminance characteristics even after 1000 cycles of repeated bending/relaxation, however, the devices based on the flexible metal grid TCs and ITO-coated PET exhibit degraded performances under the same conditions (*r* and applied voltage), as shown in Fig. 5f.

## Discussion

In summary, we introduced the fabrication of the metal grid structures based on self-pinning assisted direct inking of silver ions, and the demonstrated the SMN treatment for transforming the loose and weak NP networks of the metal grid structures to compact and robust metallic structures. SMN led to not only decreasing *R*_s_ of the SG metal grid TCs without degrading *T*_550nm_, but also enhancing the electromechanical stability of the flexible metal grid under static bending, folding and dynamic bending stresses. SMN can be used with chemical or photonic sintering to enhance optoelectrical properties and electromechanical stability of various metallic structures fabricated on flexible substrates in roll-to-roll system. Also, SMN facilitates the reinforcement of optoelectrical properties and electromechanical stability of random loose micro/nanoscale metallic structures. This method is particularly a promising candidate for producing next-generation optoelectronic and micro/nanoelectronic devices based on advanced metallic structures with high transparency and conductivity, reinforced mechanical strength and flexibility.

## Methods

### Materials

The silver ionic ink (TEC-IJ-060, Inktec) with a metal concentration of 12 wt%, consisted of silver ions (silver alkyl carbamate complexes), a base solvent (methanol and toluene), and additives. Before self-pinning assisted direct inking of silver ions, the silver ionic ink of 0.2 ml was heated at 100 °C for 5 min to evaporate the base solvent. The silver alkyl carbamate complexes were decomposed to silver NPs, carbon dioxide, and the corresponding alkyl amines by heating of 60 °C for a few minutes^33^.

### Fabrication of the PDMS mold

The PDMS solution (Sylgard 184, Dow Corning) was formed by mixing the silicon elastomer kit and a curing agent (10:1), and this mixture was poured onto a SU-8 master. After PDMS curing at 100 °C for 1 hour, the PDMS replica of the grid-patterned mold was carefully released from the SU-8 master. The grid-patterned cavity was designed with a width (*w*) of 3, 5, 10, 15 μm and a spacing (*s*) by considering the geometrical shadow zone, *T* = *s*^2^/(*s* + *w*)^2^. The value of *h* is a half of *w* to prevent destruction of the silver NP structures during the detachment of PDMS mold.

### Self-pinning assisted direct inking

The silver ionic ink (~10 μL) was dispensed onto a glass substrate. It was squeezed and filled inside a grid-patterned mold under low pressures (*P* < 120 kPa) and low temperatures (*T* = 60 °C). During solvent evaporation, some of the silver ions were thermally decomposed to silver NPs. Even after the mold detachment, the grid-patterned ink was maintained due to packings of silver NPs at contact lines of the meniscus. The grid-patterned ink was completely decomposed at heating of 150 °C to eliminate large amounts of organic complexes derived from the thermal decomposition and to improve the thermal decomposition rate of the silver ions. The silver NP structures were sintered at *T*_S_ = 200–300 °C for 1 min.

### Selective multi-nanosoldering

The SN process consisted of an electroless plating, a cleaning process and a sintering process. The electroless plating was used to generate the SG silver NPs on the metal grid TCs from the reaction of a solution (1:1.6) containing silver nitrate (Tollen’s reagent) and a reducing sugar (glucose) at 30 °C. After the cleaning process, the SG silver NPs were merged with the metal grid structures by a thermal sintering.

### Transfer process

A glass substrate (sodalime) was treated using 1 H, 1 H, 2 H, 2H-perfluorooctyl-trichlorosilane (448931, Sigma-Aldrich) for 20 min in a vacuum chamber. The SG metal grid TCs on the glass substrate were re-treated with the fluorinated silane for 5 min. The NOA 81 solution was poured onto the SG metal grid TCs on the glass substrate, and it was covered by another fluorinated glass substrate (borosilicate) to generate a uniform, thin and transparent NOA 81 film (below 200 μm). A thickness of the NOA 81 films was controlled using a stack of 3 M magic tape. The NOA 81 solution were cured to the NOA 81 film under UV exposure. After the glass substrate (borosilicate) was firstly detached from the NOA 81 film due to relatively weak adhesion energy, the flexible metal grid TCs were separated from the glass substrate (sodalime).

### Fabrication of flexible organic light-emitting diodes

On top of the SG metal grid TCs, highly conductive PEODT:PSS (Clevios PH 1000) was spin-coated with 3000 rpm for 30 sec, and annealed them at 100 °C on the hot plate for 10 min. After coating the PH1000, the low conductive PEDOT:PSS (Clevios PVP AI4083) as a hole injection layer was coated with 2500 rpm for 30 sec on the SG metal grid TCs as well as ITO pre-coated (150 nm) PET as a control device. After coating the PEDOE:PSS, samples were loaded into a thermal evaporator to deposite the organic multilayers, inorganic buffer layers and a metal electrode under high vacuum condition (2 ×10^−6^ Torr) with below configuration: MoO_3_ (10 nm)/4,4′-Bis(N-carbazolyl)-1,1′-biphenyl (CBP, 50 nm)/CBP doped with bis(2-phenylpyridine)iridium(III)-acetylacetonate (Ir(ppy)_2_acac, 7 wt%) (20 nm)/2,2,2″-(1,3,5-Benzinetriyl)-tris(1-phenyl-1-H-benzimidazole) (TPBi, 60 nm)/LiF (1 nm)/Al (100 nm).

### Characterization

The SG metal grid TCs were imaged using field-emission SEM (S-4800, Hitachi). Cross-sectional profiles of the SG metal grid TCs were measured using FIB-SEM (Helios Nanolab 600, FEI), and the surface roughness of the SG metal grid TCs was measured using AFM (XE-100, Park Systems). The transmittance spectra were measured using a UV-VIS-NIR spectrophotometer (Lambda 1050, Perkin-Elmer). The *R*_s_ of the SG metal grid TCs was measured using the two-terminal method and four-point probe method (4200-SCS, Keithley). Two electrodes between the metal grids, separated by a square area (25 mm^2^), were fabricated using conductive pens of CW2200MTP and CW2900 (ITW Chemtronics). The electro-luminance characteristics of OLEDs were measured using a source meter (2400, Keithley), a fiber-optic spectrometer (EPP2000, StellarNet) and a calibrated Si-photodiode (FDS-100, Thorlab) in N2-filled glove box. The motorized goniometric system was used to characterize angular electroluminescence profile of OLEDs. The cyclic bending test of OLEDs was performed using a custom-made bending tester that can control the number of cycles and a bending radius.

## Supplementary information

Supplementary information.
