# Supplementary material for: Selective multi-nanosoldering for fabrication of advanced solution-processed micro/nanoscale metal grid structures
Source: Sci Rep. 2020 Apr 22;10:6782. doi: 10.1038/s41598-020-63695-0 (PMC7176656; doi:10.1038/s41598-020-63695-0)
Supplement: Supplementary file 1 — Supplementary information. [file 41598_2020_63695_MOESM1_ESM.docx]

Selective multi-nanosoldering for fabrication of advanced solution-processed micro/nanoscale metal grid structures

Y.S. Oh,^a^ J. Lee,^b^ D.Y. Choi,^a^ H. Lee,^b^ K. Kang,^a^ S. Yoo,^b^ I. Park^a^* and H.J. Sung^a^*

*^a^Department of Mechanical Engineering, KAIST, 291 Daehak-ro, Yuseong-gu, Daejeon 34141, Korea, E-mail:* [*inkyu@kaist.ac.kr*](mailto:inkyu@kaist.ac.kr)*, E-mail:* [*hjsung@kaist.ac.kr*](mailto:hjsung@kaist.ac.kr)

*^b^School of Electrical Engineering, KAIST, 291 Daehak-ro, Yuseong-gu, Daejeon 34141, Korea*


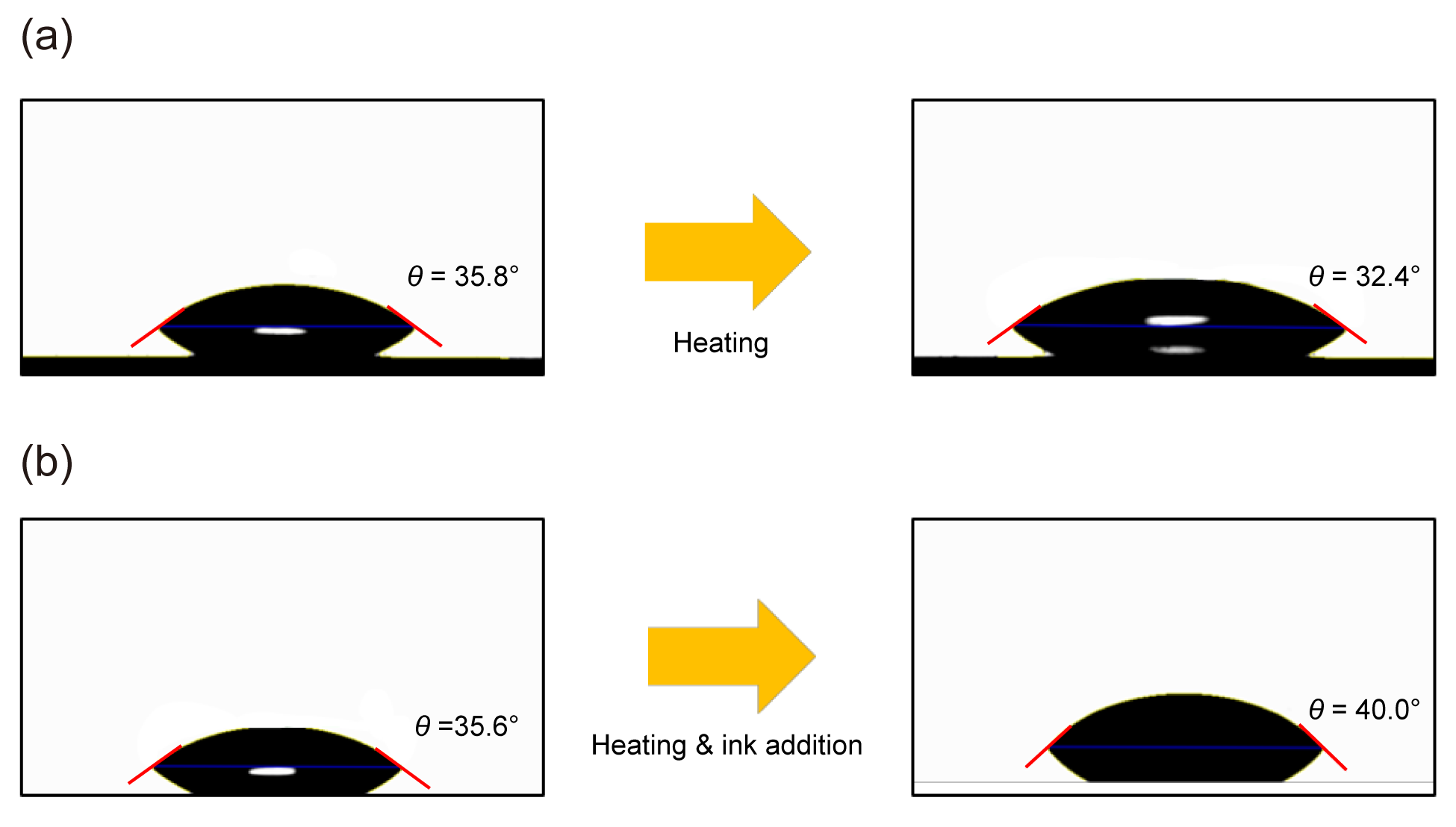


**Figure S1.** Photographic images for the measured *θ*_E_ of the ink droplet (a) after heating at 60 °C for 2 min, and (b) after heating and ink addition.

The measured *θ*_E_ of the ink droplet after ink addition and heating is shown in Figure S1. An ink volume of 5 μl was added to the ink droplet (5 μl). The measured *θ*_E_ of the ink droplet without heating showed similar (or slightly decreased) valuses due to solvent evaporation. After heating at 60°C for 2 min, the ink droplet showed a spreading behavior at *θ*_E_ = 50°. The measured *θ*_E_ of the ink droplet increased with an addition of ink due to the thermal reduction of silver ions. The measured *θ*_E_ of the ink droplet slowly decreased with solvent evaporation.


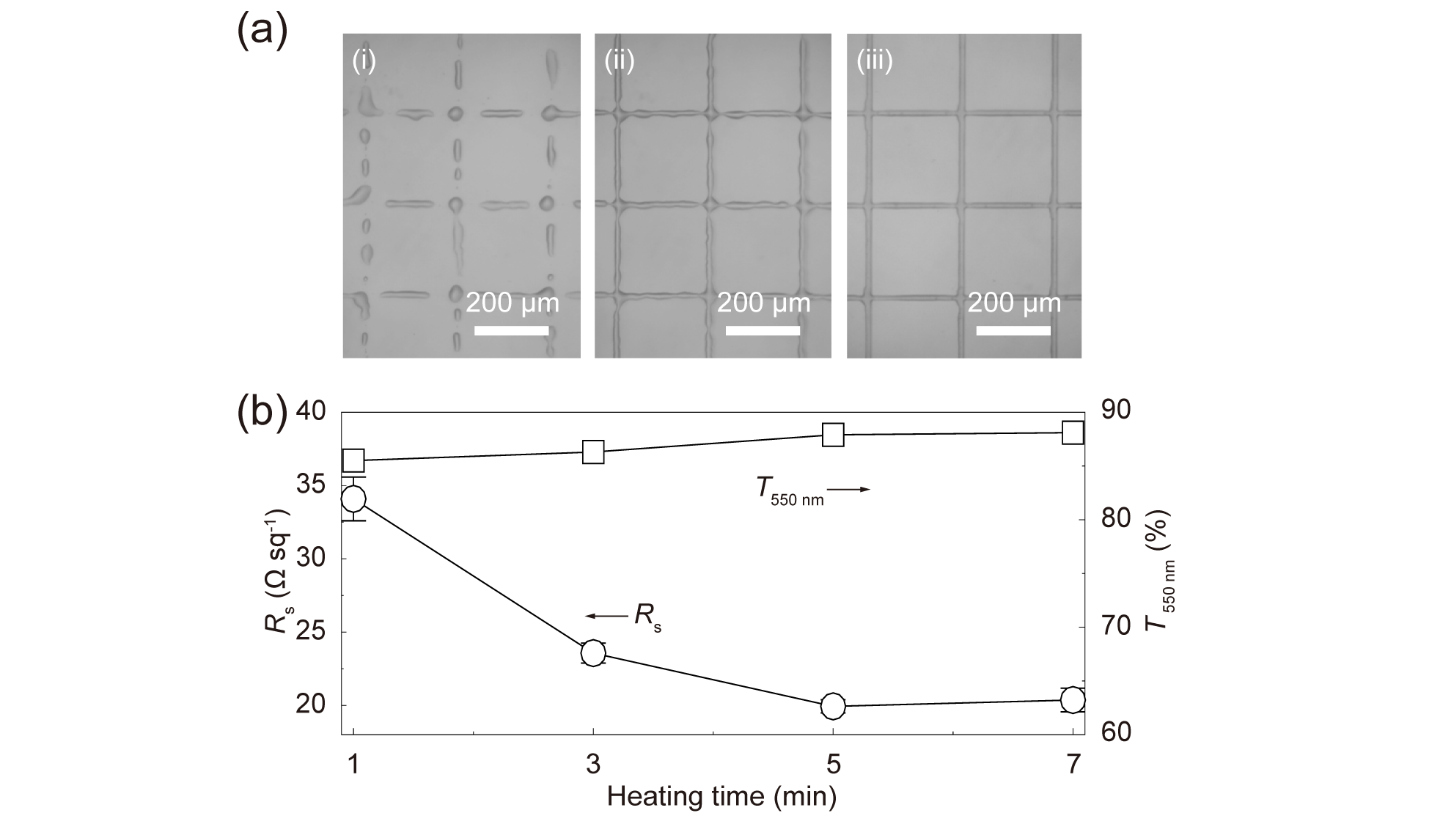


**Figure S2.** (a) A wetting behavior of silver ionic ink after the mold detachment at different heating times (b) Sheet resistance and transmittances of the SP metal grid TCs after the mold detachment at different heating times.

Figure S2a shows a wetting behavior of silver ionic ink after the mold detachment at a heating time of (i) 30 sec, (ii) 60 sec and (iii) 300 sec, respectively. These results mean that a wetting behavior of the grid-patterned ink can be controlled by increasing a heating time due to the deposition of silver NP and organic complexes at contact lines. Figure S2b shows *R*_s_ and *T*_550nm_ of the SP metal grid TCs fabricated at different times of the mold detachment.


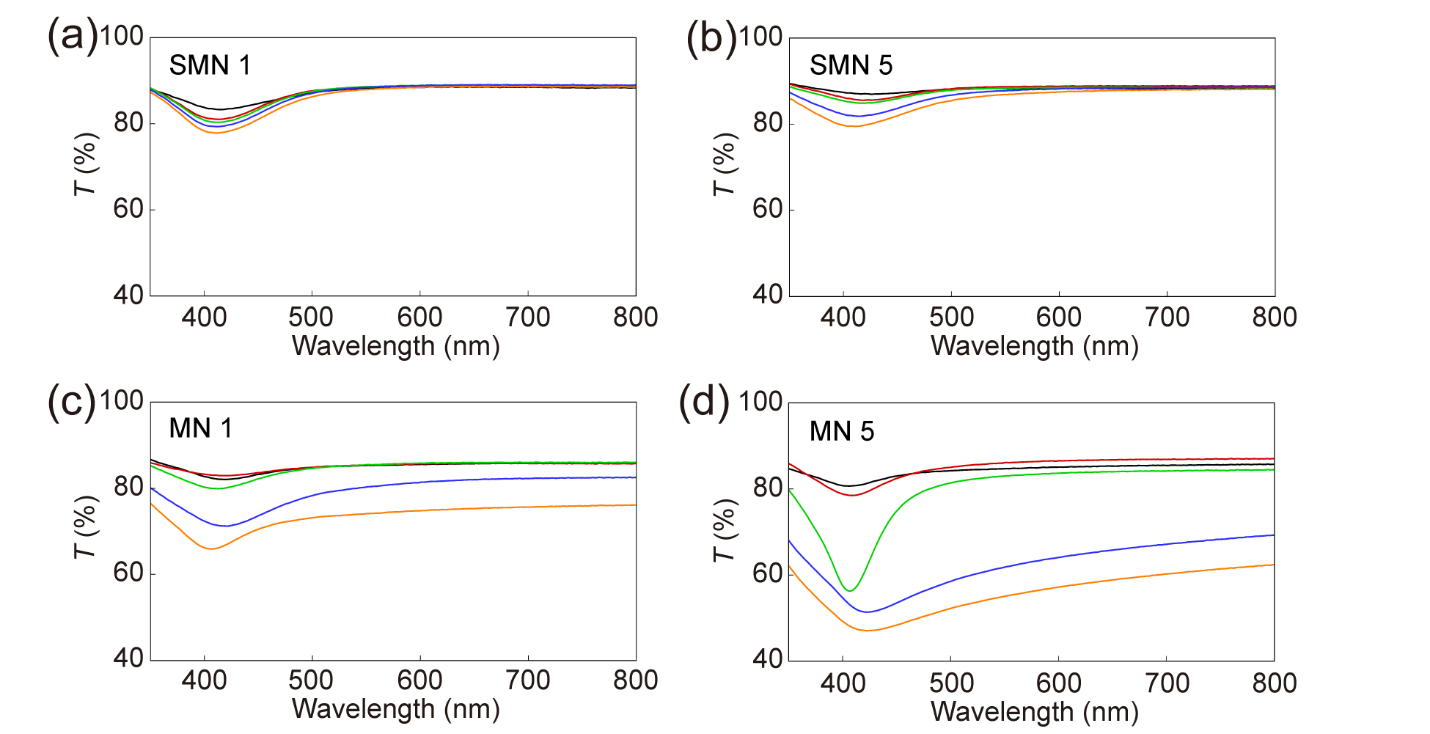


**Figure S3.** Transmittance spectra of the SG-SP metal grid TCs generated using (a) SMN 1; (b) SMN 5; (c) MN 1; and (d) MN 5, repectively.

Figure S3 shows transmittance spectra over a wavelength range of 350-800 nm of the SG-SP metal grid TCs during SMN 1, SMN 5, MN 1 and MN 5, respectively. The spectral intensity for 350-800 nm decreased at MN 1 of 3 cycles and MN 5 of 2 cycles, while it was not observed during SMN 1 and 5 of 4 cycles, respectively.
